# P-523. Impact of Tailored Education on Engagement in Sexual Health and HIV Prevention Services Among US Women

**DOI:** 10.1093/ofid/ofae631.722

**Published:** 2025-01-29

**Authors:** Anandi N Sheth, Shaina Y Vincent, Laura Simone, Chris Napolitan, Jeffrey D Carter, Melissa Rodriguez, Jenniffer A Meza Jimenez, Leah Molloy

**Affiliations:** Emory University School of Medicine, Atlanta, Georgia; PRIME Education, Shelby Twp, Michigan; PRIME Education, LLC, Fort Lauderdale, Florida; PRIME Education, Shelby Twp, Michigan; PRIME Education, LLC, Fort Lauderdale, Florida; PRIME Education, Shelby Twp, Michigan; PRIME Education, Shelby Twp, Michigan; PRIME Education, Shelby Twp, Michigan

## Abstract

**Background:**

Women in the US are facing increasing barriers to sexual health services. This project aimed to enhance sexual health literacy and linkage to HIV prevention among US women.

**Methods:**

From 6/2023-8/2023, six patient education sessions on sexual health and HIV prevention were held in women’s health clinics in California, Georgia, and Virginia. Surveys were given to participating patients and healthcare professionals (HCPs) before/after each session and one month later. Content was shared nationally through an online toolkit for HCPs and a public social media video.

**Results:**

Among the 88 patients who attended a sexual health education session (mean age: 44 years; 60% Black, 83% previously tested for HIV), baseline awareness of pre-exposure prophylaxis (PrEP) (23%) and resources for HIV testing/prevention (63%) significantly increased to 71% (*P* < .001) and 84% (*P* = .002), respectively, after the sessions. More women felt confident in HIV prevention after the education than before (93% vs. 82%, *P* = .028). After education, all participating HCPs (n = 6) correctly identified that a woman’s HIV risk is nearly constant throughout life and that PrEP should be offered to anyone who asks for it, compared to 50% correctly answering each question at baseline (*P* = .181). When asked about patients’ barriers to HIV/STI testing, HCPs most frequently chose fear of judgment (50%), while patients reported cost concerns (27%) and test-related anxiety (26%). When asked what would make testing easier, learning about free or low-cost local testing centers was most frequently chosen by both patients (49%) and HCPs (67%). Discordances were observed regarding PrEP barriers. The top patient-reported barrier was insurance coverage/medication costs (48%), while HCPs perceived fear of side effects as the main barrier (67%). One month later, 73% (n = 19/26) of patients had gotten tested for HIV, and HCPs had discussed HIV screening/prevention with 94 women and helped 38 start PrEP. As of 1/2024, the online toolkit and social media video were accessed by 1,057 and 4,054 people, respectively.

**Conclusion:**

This tailored educational initiative offered insights into women’s barriers to HIV screening and prevention, after which women’s participation in HIV testing and awareness of PrEP improved.

**Disclosures:**

**All Authors**: No reported disclosures

